# Expressions of heparanase and upstream stimulatory factor in hepatocellular carcinoma

**DOI:** 10.1186/s40001-014-0045-9

**Published:** 2014-08-23

**Authors:** Bin Chen, Xiao-Peng Chen, Ming-Shi Wu, Wei Cui, Min Zhong

**Affiliations:** 1 Department of General Surgery, Affiliated Yijishan Hospital of Wannan Medical College, Wuhu 241001, Anhui Province, China; 2 Central Laboratory, Affiliated Yijishan Hospital of Wannan Medical College, Wuhu 241001, Anhui Province, China

**Keywords:** Heparanase, Upstream stimulatory factor, Hepatocellular carcinoma, Recurrence

## Abstract

**Background:**

The expression of heparanase (HPSE) was associated with postoperative metastatic recurrence in patients with hepatocellular carcinoma (HCC). The six E-box binding sites in the core promoter of the *HPSE* gene suggested that transcription factors of E-box such as upstream stimulatory factor (USF) might regulate the transcription of the *HPSE* gene. The aim of our study is to measure the levels of HPSE and USF expression and investigate the relationship between USF expression and clinicopathological parameters in patients with HCC.

**Methods:**

HPSE, USF1 and USF2 expressions in human HCC cell lines (BEL-7402, HepG2 and HCCLM3) and 15 fresh human HCC tissue samples were measured by real-time reverse transcriptase-PCR and Western blot analysis. The normal liver cell line QSG7701 or fresh normal liver tissue samples obtained from 15 additional surgical patients with hepatic rupture was used as a control. The protein expressions were determined by immunohistochemistry in paraffin-embedded human HCC tissues and corresponding non-neoplastic tumor surrounding tissues (NTST) of 57 patients.

**Results:**

HPSE, USF1 and USF2 mRNA expressions were increased in HCC cell lines and HCC tissues compared with normal liver cell line and normal liver tissue. The protein expressions of HPSE, USF1 and USF2 in HCC cell lines and HCC tissues were also increased. Both USF1 and USF2 expressions were positively correlated with HPSE. USF1 and USF2 expressions were increased in patients with liver cirrhosis, worse tissue differentiation, advanced HCC stages and metastatic recurrence.

**Conclusions:**

Increased USF in HCC is associated with HPSE expression. USF might be an important factor in regulating HPSE expression and act as a novel marker of metastatic recurrence of HCC patients.

## Background

Hepatocellular carcinoma (HCC) is one of the most common human malignancies and responsible for approximately 5% of all cancer-related deaths in the world. Postoperative metastatic recurrence is now known to be the major cause of death of patients with HCC [1]–[3].

Metastatic recurrence of HCC is a complex and multistep biological process that includes loss of adhesion, migration, invasion and proliferation of cancer cells. Many molecules contribute to this process, and some of these molecules are involved in the mechanical aspects, whereas others modulate regulatory pathways.

Heparanase (HPSE) is an endo-beta-glucuronidase capable of cleaving heparan sulfate. HPSE plays an important role in extravasation and invasion of tumor cells by cleaving heparan sulfate side chains of heparan sulfate proteoglycans on cell surfaces and in extracellular matrices of basement membrane [4]–[6]. Previous studies demonstrated that increased HPSE activity has been detected in various tumors and was found to correlate with their metastatic potentials [7]–[11]. Therefore, HPSE might be a metastatic marker and predict postoperative metastatic recurrence in patients with HCC.

To understand the mechanisms of HPSE up-regulation in tumors, the promoter sequence of HPSE gene was cloned and the transcription factor binding sites (TFBS) were subsequently analyzed. Besides various TFBS such as specificity protein 1 (Sp1), GA-binding protein (GABP) and epidermal growth factor-1 (EGR-1) as described in the literatures [12]–[14], the core promoter included six E-box binding sites which were located at 553-548, 542-537, 402-397, 286-281, 34-29 and 14-9 bp upstream of the ATG translation initiation site, respectively [15]. The results suggested that HPSE gene expression might be regulated by E-box sites.

The transcription factor upstream stimulatory factor (USF) was originally identified in HeLa cells by biochemical analysis. Two different genes encode USF which had been defined as USF1 and USF2 [16]. The human cDNA cloning of USF1 and USF2 revealed that the USF belongs to the c-Myc related family of DNA-binding proteins which have a helix-loop-helix motif and a leucine repeat, and that USF interacts with its target DNA sequence (E-box) as a dimer and regulates the transcription of numerous genes [17].

However, the expression of USF1 and USF2 in HCC is unknown. The relationship between USF expression and clinicopathological parameters in patients with HCC remains to be determined. The aims of our study are to measure expression of USF in HCC and evaluate the relationship between USF expression and clinicopathological parameters in patients with HCC.

## Methods

### Cell culture

Human normal liver cell line QSG7701, hepatoma carcinoma cell line BEL-7402 and HepG2 were purchased from Cell Bank (National Academy of Science of China, Shanghai, China). Human highly metastatic liver cancer cell line HCCLM3 was purchased from Liver Cancer Institute (Zhongshan Hospital, Fudan University, Shanghai, China).

QSG7701, BEL-7402, HepG2, and HCCLM3 cells were cultured in DMEM/GlutaMax-1 (Invitrogen, Shanghai, China) supplemented with 10% FBS (Life Technologies, Carlsbad, CA, USA), 100 IU/ml penicillin and 100 mg/ml streptomycin under a 5% CO_2_ atmosphere at 37°C.

### Patients and tissue selection

Fifty-seven patients (44 males and 13 females) underwent primary surgical resection for HCC in Yijishan Hospital of Wannan Medical College (Anhui province of China) between January 2008 and December 2011. The average age was 55 years (range from 32 to 82 years). None of the patients had received preoperative therapy. All primary HCC tissue samples taken from the 57 patients were fixed in 10% formalin, embedded in paraffin, sectioned consecutively at 4 μm and stained by H&E for immunohistochemical analysis. The diagnoses were confirmed by histopathologic study. Tumor stage was determined according to the 2002 International Union Against Cancer TNM classification system. Tumor differentiation was graded by the Edmondson grading system. The patients were divided into high-tendency to metastatic recurrence group (n = 35) and low-tendency to metastatic recurrence group (n = 22) according to the presence or absence of the cancer emboli, intrahepatic dissemination (satellite foci or multiple nodules), disintegrated tumor capsule and/or lymph node metastasis. The clinicopathological features of these patients are summarized in Table 1.

**Table 1:** Relationship between heparanase (HPSE), upstream stimulatory factor (USF) expression and tumor characteristics of hepatocellular carcinoma (HCC)

**Features**	**n**	**HPSE expression**			**USF1 expression**			**USF2 expression**		
		**+**	**−**	***P***	**+**	**−**	***P***	**+**	**−**	***P***
Gender										
Male	44	31	13	0.789	25	19	0.850	27	17	0.991
Female	13	8	5		7	6		8	5	
Age (years)										
< 55	39	28	11	0.850	22	17	0.952	26	13	0.230
≥ 55	18	11	7		10	8		9	9	
Tumor diameter (cm)										
< 5	18	11	7	0.419	7	11	0.672	8/1	10	0.520
≥ 5	39	28	11		18	21		21	18	
AFP (μg/l)										
< 400	21	25	11	0.709	13	8	0.503	12	9	0.614
≥ 400	36	14	7		19	17		23	13	
HBsAg										
Positive	41	30	11	0.217	20	21	0.073	24	17	0.477
Negative	16	9	7		12	4		11	5	
Cirrhosis										
Yes	21	13	8	0.419	16	5	0.020	17	4	0.021
No	36	26	10		16	20		18	18	
Histological differentiation										
Well + moderate	38	22	16	0.016	17	21	0.014	19	19	0.012
Poor	19	17	2		15	4		16	3	
TNM staging										
I + II	31	17	14	0.016	12	19	0.004	15	16	0.028
III + IV	26	22	4		20	6		20	6	
Tendency to MR										
High	35	29	6	0.003	24	11	0.017	26	9	0.012
Low	22	10	12		8	14		9	13	
Postoperative MR										
Yes	21	18	3	0.032	14	7	0.035	16	5	0.002
No	30	21	15		12	18		6	24	
HPSE expression										
Positive	39				27	12	0.003	28	11	0.018
Negative	18				5	13		7	11	

*Abbreviations*: *AFP* alpha-fetoprotein, *HBsAG* hepatitis B surface antigen, *MR* metastatic recurrence.

Fifteen patients were selected randomly from the 57 cases. The fresh tissue samples were immediately frozen in liquid nitrogen and stored at −80°C after hepatectomy for real-time RT-PCR and Western blotting. Non-neoplastic tumor surrounding tissues (NTST) specimens were obtained from tissues at a clear distance from the tumor edge (>1 cm). The fresh normal liver tissue (NLT) was obtained from 15 additional surgical patients with hepatic rupture.

Written informed consent was obtained from these patients. The research protocol followed the ethical guidelines of the Declaration of Helsinki and was approved by the Ethics Committee of the Yijishan Hospital.

### RNA isolation and real-time RT-PCR analysis

The extraction of total RNA was performed by using Trizol solution (Invitrogen, Shanghai, China) according to the manufacturer’s protocols. The RNA was quantified by using UV spectrophotometry at 260 nm. The RNA specimens were stored at −80°C until real-time RT-PCR. Reverse transcription was performed with the Moloney murine leukemia virus (M-MLV) reverse transcriptase (Promega, Beijing, China), using 2 μg of total RNA and following the manufacturer’s instructions. The resulting cDNA was used for real-time quantitative PCR detection. Glyceraldehyde-3-phosphate dehydrogenase (GAPDH) was used as an internal control.

PCR was performed in 25 μl of reaction mixture containing 100 ng of both sense and antisense primers (Shanghai Shenggong Company, Shanghai, China), 1 μl (200 ng) of the cDNA and 12.5 μl SYBR Green PCR Master Mix (QIAGEN SA). The sequences of the primers used for PCR are listed in Table 2. Negative controls (PCR mix without cDNA) were included. The thermal cycle was 10 minutes at 95°C, and 40 cycles of 95°C for 15 seconds, 55°C for 30 seconds and 72°C for 30 seconds. All assays were evaluated using the Stepone plus™ real-time PCR system (ABI Company, USA), and relative expression was calculated by normalizing the Ct (threshold cycle) of the target gene to the Ct of the GAPDH housekeeping gene in the same sample. The experiments, including RNA isolation, reverse transcription and real-time quantitative PCR, were performed in triplicate.

**Table 2:** The sequences of the primers used for PCR

**Genes**	**Primers**	**Sequences**	**Size of product (bp)**
*HPSE*	Forward	5′- GCACAAACACTGACAATCCAAG -3′	101
	Reverse	5′- AAAAGGATAGGGTAACCGCAA -3′	
*USF1*	Forward	5′- TTGTCCTGTGCTTGCTTAGAGT -3′	102
	Reverse	5′- CAGGGAAAGGAAGAACCAATG -3′	
*USF2*	Forward	5′- AAATTGATGGAACCAGAACACC -3′	136
	Reverse	5′- TTGTCTGCGTTACAGTCTGGAAT -3′	
*GAPDH*	Forward	5′- GTGGTCTCCTCTGACTTCAACA -3′	136
	Reverse	5′- CCACCACCCTGTTGCTGTAG A -3′	

### Western blot analysis

Total protein extracts (50 μg) were prepared in sample buffer containing 20 mM Tris (pH 8.0), 5 mM ethylenediaminetetraacetic acid (EDTA), 0.5% Triton X-100 and complete Mini, EDTA-free protease inhibitors (1:25; Roche Diagnostics, Mannheim, Germany).

For Western blot analyses, 50 μg of protein were loaded on a 12% SDS-PAGE gel. The gel was transferred onto a nitrocellulose transfer membrane (Santa Cruz Biotechnology Inc., Santa Cruz, CA, USA) following separation. Rabbit-anti-HPSE polyclonal antiserum (Jinqiao biotechnology, Beijing, China; at 1:250 dilution), rabbit-anti-USF1 polyclonal antiserum (Jinqiao biotechnology, Beijing, China; at 1:250 dilution), rabbit-anti-USF2 polyclonal antiserum (Santa Cruz Biotechnology Inc, Santa Cruz, USA; at 1:250 dilution) or rabbit-anti-GAPDH polyclonal antiserum (Santa Cruz Biotechnology Inc., Santa Cruz, CA, USA; at 1:10,000 dilution) were used as primary antibody, respectively. Horseradish peroxidase (HRP)-conjugated anti-rabbit IgG (Maixin biotechnology, Fujian, China) was used as secondary antibody at 1:10,000 dilution. Protein bands were visualized using Chemiluminescence Imaging Systems (Cell Signaling Technology, Danvers, MA, USA). The band intensity was measured using Quantity One software (Bio-Rad Laboratories, Hercules, CA, USA). GAPDH was used as a loading control.

### Immunohistochemistry

The HCC tissue sections of 57 patients were deparaffinized and rehydrated. Then, the sections were boiled in EDTA (1 mmol/l; pH 8.0) for antigen epitope retrieval. Endogenous peroxidase was blocked with 0.3% hydrogen peroxide. After rinsing, slides were incubated with a mouse polyclonal antibody against HPSE (Jinqiao biotechnology, Beijing, China; at 1:500 dilution), USF1 (Jinqiao biotechnology, Beijing, China; at 1:100 dilution) or USF2 (Santa Cruz Biotechnology Inc., Santa Cruz, USA; at 1:300 dilution) overnight at 4°C. After washing, the slides were incubated for 30 minutes with HRP-conjugated secondary antibody (Maixin biotechnology, Fujian, China) at room temperature. After this incubation, the slides were washed three times in PBS, and the antibody complexes were colored with diaminobenzidine (DAB) and then counterstained with hematoxylin. The total HPSE and USF immunostaining score was calculated as the sum of the presence of positively stained tumor cells and the staining intensity. Briefly, the percent positive staining was scored as 0 (<5%, negative), 1 (5 to 25%, sporadic), 2 (25 to 50%, focal), 3 (50 to 75%, diffuse) or 4 (>75%, diffuse). The staining intensity was score as 0 (no staining), 1 (weakly stained), 2 (moderately stained) or 3 (strongly stained). Each specimen was evaluated in eight fields at × 400 magnification by two independent pathologists who were unaware of the clinical data. Both percent positivity of cells and staining intensity were decided under double-blind condition. The total immunostaining score was calculated with the value of percent positivity score × staining intensity score, which ranged from 0 to 12. We defined HPSE, USF1 and USF2 expression levels as follows: – (score 0 to 1), + (score 2 to 3), ++ (score 4 to 5) and +++ (score > 5). For inconsistent evaluations of individual slides, both observers reviewed the slide again to obtain a consensus. The patients were divided into negative expression group (–) and positive expression group (+, ++ and +++) according to HPSE or USF expression level.

### Follow-up

All patients were followed up until December 2012. The median follow-up time of patients with HCC was 23 months (range from 12 to 60 months). Recurrence and metastasis were diagnosed by clinical examination, alpha-fetoprotein (AFP) measurement, liver ultrasonography, and computed tomography (CT) scan.

### Statistical analysis

The results of the descriptive analyses of variables were reported as mean ± standard deviation (SD). Analysis of variance and q-test were used to compare the difference among/between groups. Statistical comparisons for significance between different groups were evaluated by the chi-square test or the Fisher’s exact test. Spearman’s rank correlation coefficient was used to find the correlation between two sets of data. All statistical analyses were performed using the SPSS 13.0 software package (SPSS Inc., Chicago, IL, USA), and *P* < 0.05 was considered statistically significant.

## Results

### Expressions of HPSE, USF1 and USF2 in liver cancer cell lines and primary HCC

**Figure 1:**
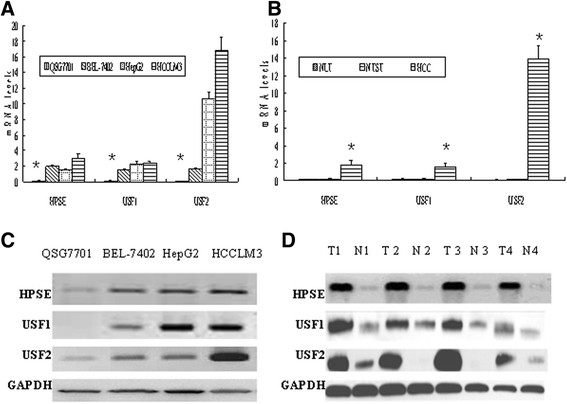
**Heparanase (HPSE), upstream stimulatory factor (USF)1 and USF2 mRNA and protein expressions in hepatocellular carcinoma (HCC).** HPSE, USF1 and USF2 mRNA and protein expressions were significantly increased in human HCC cell lines and primary HCC surgical specimens assessed with RT-PCR or Western blot. **(A)** HPSE, USF1 and USF2 mRNA expressions in human HCC cell lines. The normal liver cell line QSG7701 was used as a control. *Compared with BEL-7402, HepG2 and HCCLM3, *P* < 0.01. **(B)** HPSE, USF1 and USF2 mRNA expression in normal liver tissue, human primary HCC tumor tissues and adjacent non-neoplastic tumor surrounding tissues (NTST) (n = 15). *Compared with NLT and NTST, *P* < 0.01. **(C)** HPSE, USF1 and USF2 protein expressions in human HCC cell lines; **(D)** HPSE, USF1 and USF2 protein expressions in four representative human primary HCC tumor tissues (T) and adjacent non-tumor liver tissues (N). All experiments were performed three times.

The mean levels of HPSE, USF1 and USF2 mRNA expression in the three HCC cell lines were significantly higher than those in normal liver cell line QSG7701 (*P* < 0.05). Furthermore, USF2 expression in HCCLM3 was significantly higher than those in BEL-7402 and HepG2 (*P* < 0.05) (Table 3, Figure 1A). The mean level of HPSE mRNA expression in HCC tumor tissues was significantly increased compared with corresponding NTST and healthy NLT (all *P* < 0.01, respectively). USF1 and USF2 were also found to be up-regulated in primary HCC tumor tissues (all *P* < 0.001, respectively) (Table 3, Figure 1B).

**Table 3:** The relative mRNA expression levels of heparanase (HPSE), upstream stimulatory factor (USF)1 and USF2 in human hepatocellular carcinoma (HCC) cell lines and HCC tissues

**Genes**	**Cells**				**Tissues**		
	**QSG7701**	**BEL-7402**	**HepG2**	**HCCLM3**	**NLT**	**NTST**	**HCC**
*HPSE*	0.11 ± 0.06^a^	1.98 ± 0.13	1.50 ± 0.14	3.01 ± 0.65	0.15 ± 0.08	0.20 ± 0.09	1.75 ± 0.55^c^
*USF1*	0.11 ± 0.05^a^	1.50 ± 0.12	2.34 ± 0.32	2.38 ± 0.23	0.18 ± 0.08	0.22 ± 0.07	1.61 ± 0.45^c^
*USF2*	0.10 ± 0.04^a^	1.70 ± 0.14	10.61 ± 0.92^b^	16.84 ± 1.66^b^	0.12 ± 0.09	0.14 ± 0.09	13.92 ± 1.45^c^

*Abbreviations*: *HCC* hepatocellular carcinoma, *NLT* normal liver tissues, *NTST* non-neoplastic tumor surrounding tissue. ^a^Compared with BEL-7402, HepG2 and HCCLM3, *P* < 0.01; ^b^Compared with BEL-7402, *P* < 0.01; ^c^Compared with NLT and NTST, *P* < 0.01.

The levels of HPSE, USF1 and USF2 protein expression in HCC cell lines were significantly increased (Figure 1C). The HPSE, USF1 and USF2 protein expression levels were all increased in HCC tissues compared with corresponding NTST (Figure 1D).

### Immunohistochemical staining of HPSE, USF1 and USF2 in HCC

**Figure 2:**
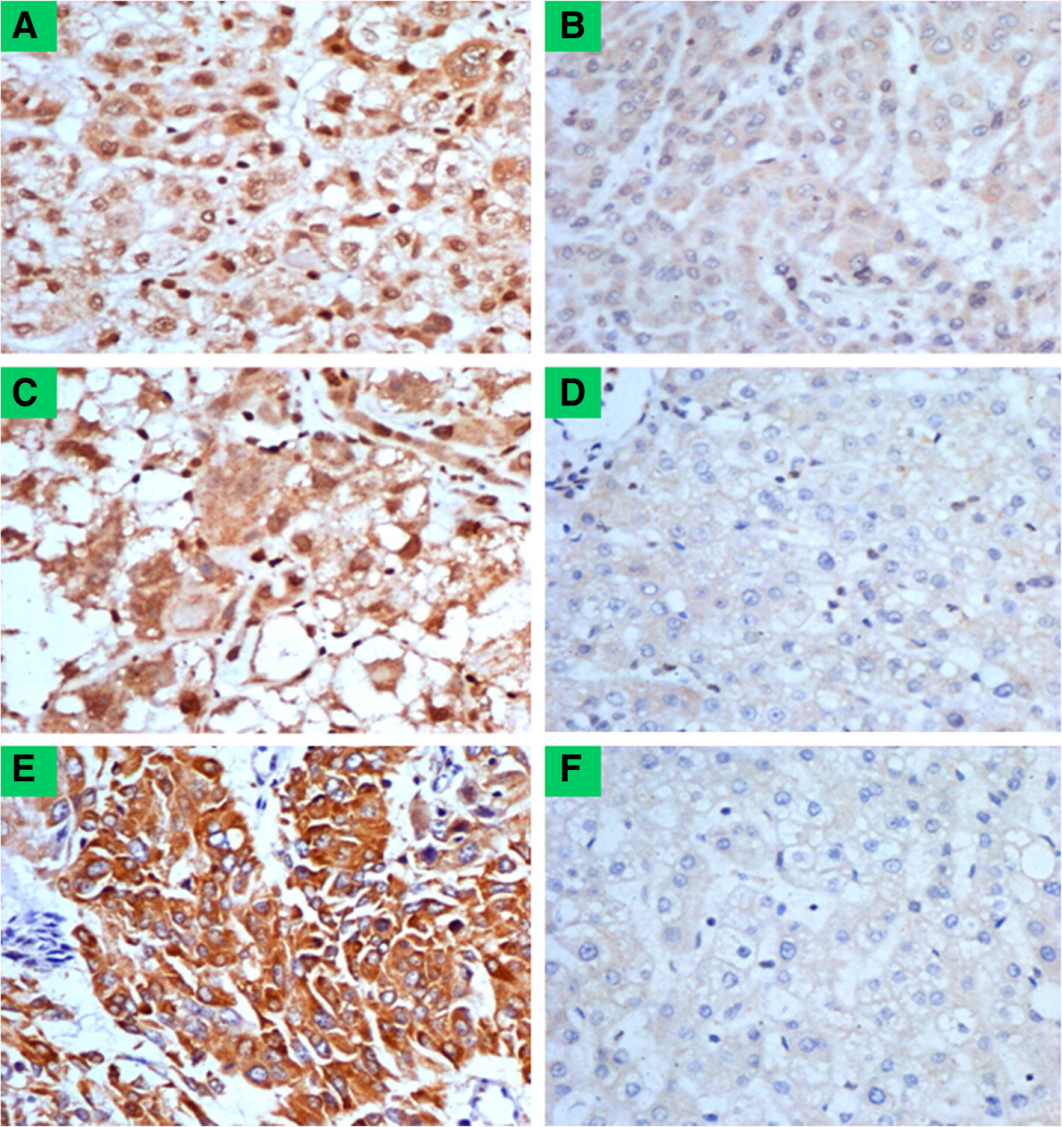
**Heparanase (HPSE), upstream stimulatory factor (USF)1 and USF2 protein expression in primary hepatocellular carcinoma (HCC) tissues.** HPSE, USF1 and USF2 protein expressions were significantly increased in primary HCC surgical specimens (n = 57) detected by immunohistochemistry (original magnification × 400). **(A)** HPSE-positive expression in HCC tumor tissues. Positive signal of HPSE was brown-yellow granules in nuclei of HCC cells. **(B)** HPSE weakly positive expression in corresponding non-neoplastic tumor surrounding tissues (NTST). **(C)** USF1-positive expression in HCC tumor tissues. Positive signal of USF1 was brown-yellow granules in nuclei of tumor cells. **(D)** USF1-negative expression in NTST. **(E)** USF2-positive expression in HCC tumor tissues. Positive signal of USF2 was yellow or brown granules in cytoplasm of HCC cells, which were distributed in dots and patches. **(F)** USF2-negative expression in NTST. All experiments were performed twice.

HPSE and USF1 staining were predominantly in the nuclei, and the positive signal was brown-yellow granules in tumor cell nuclei. HPSE and USF1 expressions in normal hepatocytes of NTST were negative or weakly positive, while mainly moderately or strongly positive in HCC cells (Figure 2A-D). There was a significant difference between the HPSE-positive rate in HCC (39/57) and that in NTST (5/57, *P* = 0.000). USF1-positive rate in HCC (32/57) was significantly higher than that in NTST (10/57, *P* = 0.000).

USF2 staining was mainly in the cytoplasm, and the positive signals were yellow or brown granules in the cytoplasm of HCC cells, which were distributed in dots and patches. USF2 expression in normal hepatocytes of NTST was negative, while mainly moderately or strongly positive in HCC cells (Figure 2E and F). There was a significant difference between USF2-positive rate in HCC (35/57) and that in NTST (9/57, *P* = 0.000).

### Relationship between protein expressions and the clinicopathological parameters

At the time of the last follow-up, 51 patients eventually achieved complete follow-up data (89.5%). Twenty-one patients were found to have postoperative metastasis or recurrence, and the other 30 were not found to have metastasis or recurrence.

HPSE protein expression was increased significantly in patients with worse tissue differentiation, advanced HCC stages, high-tendency to metastatic recurrence and postoperative metastatic recurrence. HPSE expression was not associated with sex, age, tumor size, AFP level, hepatitis B surface antigen (HBsAg) status and liver cirrhosis (*P* > 0.05) (Table 1).

USF1 and USF2 protein expressions were significantly increased in patients with liver cirrhosis, worse tissue differentiation, advanced HCC stages, high-tendency to metastatic recurrence and postoperative metastatic recurrence (*P* < 0.05). In addition, patients with positive HPSE expression had higher USF1 and USF2 expressions compared with patients with negative HPSE expression (*P* = 0.003, *P* = 0.018, respectively). Both USF1 and USF2 expressions were not associated with sex, age, tumor size, AFP level and HBsAg status (*P* < 0.05) (Table 1).

### Correlation between HPSE and USF expression

To investigate further the correlation of HPSE and USF expression in HCC, Spearman’s rank correlation coefficients were calculated. They were 0.344 (*P* = 0.009) and 0.363 (*P* = 0.005), respectively (Table 4). It suggested that both USF1 and USF2 expressions were positively correlated with HPSE expression.

**Table 4:** The correlation between heparanase (HPSE) and upstream stimulatory factor (USF) expression in hepatocellular carcinoma (HCC) tissues

**HPSE**	**USF1**				**r**	***P***	**USF2**				**r**	***P***
	**-**	**+**	**++**	**+++**			**-**	**+**	**++**	**+++**		
-	14	2	2	0	0.344	0.009	15	1	1	1	0.363	0.005
+	2	4	4	3			2	2	4	4		
++	5	2	2	6			3	4	3	5		
+++	4	1	2	4			2	3	3	4		

## Discussion

High levels of HPSE mRNA and protein are expressed in most malignant tumors including HCC and are closely associated with tumor metastasis, angiogenesis and other diverse pathological and physiological processes [4]–[11]. In our previous study, we cloned a 561-bp-long human *HPSE* gene core promoter and found it contained six E-box binding sites [15]. As one of the transcription factors of E-box sites, we speculate that transcription of the *HPSE* gene might be regulated by USF in HCC. USF is a ubiquitously expressed multifunctional transcription factor [16]–[20], but USF expression and its role in HCC remain unknown [21],[22].

In this study, we found the relative expression levels of HPSE and USF were significantly increased in the HCC cell lines and HCC tumor tissues compared with the normal liver cell line or corresponding NTST. These results suggest USF might be a biological indicator of malignant potential of HCC. Highest USF2 expression level was found in HCCLM3 among all three HCC cell lines, and HCCLM3 was a human HCC cell line with high metastatic potential [23]. Therefore, USF2 might be a potential marker of the metastatic recurrence of HCC. Furthermore, we found the up-regulations of USF1 and USF2 mRNA expressions in HCC were incompletely in line with that of HPSE expression. It might be explained that HPSE transcription was functionally regulated by many transcription factors, and USF was only one of the transcription factors [12]–[15].

In our previous study, we found that high HPSE mRNA expression was associated with worse tissue differentiation, advanced HCC stages, high-tendency to metastatic recurrence and postoperative metastatic recurrence [7]. In this study, we found the same results. Furthermore, we also found that both USF1 and USF2 expressions were significantly increased in patients with liver cirrhosis, poor differentiation, advanced tumor stages, the high-tendency to metastatic recurrence and postoperative metastatic recurrence. The close relationship between USF expression and clinicopathological features predicts that USF might boost carcinogenesis and metastatic tumor recurrence. Interestingly, USF expression, rather than HPSE, is associated with liver cirrhosis. The result suggests that USF could also play some role in the formation or regulation of liver cirrhosis.

Additionally, we found USF1 and USF2 expressions were associated with HPSE expression in HCC, and both USF1 and USF2 expressions were positively correlated with HPSE. These results suggest further HPSE expression in HCC might be regulated by USF. Of course, definite evidence and concrete mechanism remain to be further investigated.

## Conclusion

USF1 and USF2 expressions are significantly increased in HCC and positively correlated with HPSE expression. USF might be an important factor in regulating HPSE expression and act as a novel marker of metastatic recurrence of HCC patients.

## Abbreviations

AFP: alpha-fetoprotein

bp: base pair

CT: computed tomography

DAB: diaminobenzidine

DMEM: Dulbecco’s modified Eagle’s medium

EDTA: ethylenediaminetetraacetic acid

EGR-1: epidermal growth factor-1

FBS: fetal bovine serum

GABP: GA-binding protein

HBsAg: hepatitis B surface antigen

HCC: hepatocellular carcinoma

GAPDH: glyceraldehyde-3-phosphate dehydrogenase

H&E: hematoxylin and eosin

HPSE: heparanase

HRP: horseradish peroxidase

Ig: immunoglobulin

M-MLV: Moloney murine leukemia virus

NLT: normal liver tissue

NTST: non-neoplastic tumor surrounding tissues

PCR: polymerase chain reaction

Sp1: specificity protein 1

TFBS: transcription factor binding sites

USF: upstream stimulatory factor

## Competing interests

The authors declare that they have no competing interests.

## Authors’ contributions

BC and MZ carried out the RT-PCR studies and Western blot analysis. XPC conceived of the study, carried out the design and drafted the manuscript, participated in the RT-PCR study and Western blot analysis. WC performed the statistical analysis and helped to draft the manuscript. MSW carried out the immunohistochemical study. All authors read and approved the final manuscript.
